# Dupilumab improves long-term asthma control in German patients with severe asthma: Real-world data 

**DOI:** 10.5414/ALX02646E

**Published:** 2026-09-25

**Authors:** Andreas Renner, Slagjana Stoshikj, Eckard Hamelmann, Dirk Skowasch, Christian Schulz, Margret Jandl, Olaf Schmidt, Katrin Milger, Rainer Ehmann, Christian Taube, Stephanie Korn, Roland Buhl, Marco Idzko, the German Asthma Net (GAN) study group

**Affiliations:** 1 Department of Pneumology, Medical University of Vienna, Vienna, Austria,; 2 Comprehensive Center for Chest Diseases, Medical University of Vienna, Vienna, Austria,; 3 Kinderzentrum Bethel, Evangelisches Klinikum Bethel, University Bielefeld, Bielefeld,; 4 Department of Internal Medicine II - Pneumology, University Hospital Bonn, Bonn,; 5 Department of Internal Medicine II, University Hospital Regensburg, Regensburg,; 6 Hamburger Institut für Therapieforschung GmbH, Hamburg,; 7 Pneumologische Gemeinschaftspraxis und Studienzentrum KPPK, Koblenz, Germany,; 8 Division of Respiratory Medicine, Lung Research Cluster, Department of Internal Medicine, Medical University of Graz, Graz, Austria,; 9 Department of Medicine V, Ludwig-Maximilians-University (LMU) of Munich, Comprehensive Pneumology Center (CPC-M) German Center for Lung Research (DZL), Munich,; 10 Ambulante Pneumologie mit Allergiezentrum, Stuttgart,; 11 Department of Pulmonary Medicine, University Hospital Essen – Ruhrlandklinik, Essen,; 12 Department of Pulmonary and Respiratory Critical Care Medicine, Thoraxklinik Heidelberg und IKF Pneumologie Mainz, and; 13 Pulmonary Department, Mainz University Hospital, Mainz, Germany; * Contributed equally

**Keywords:** severe asthma, real-life data, monoclonal antibody treatment, biologics, airway inflammation

## Abstract

Background: Dupilumab has proven effective in severe asthma, but there is only sparse evidence on its impact on asthma control in a real-world setting. Materials and methods: All patients from the German Asthma Net (GAN) severe asthma registry who initiated dupilumab after inclusion into the registry were analyzed. Clinical effectiveness was evaluated in those with at least 2 follow-up visits, with a maximum follow-up time of 3 years. Results: A total of 481 patients with severe asthma initiated dupilumab. At baseline, median blood eosinophil count was 174 cells/µL (IQR 61.2 – 436); 184 (38.3%) were former smokers with median 9.6 pack-years (IQR 4.3 – 23.3). Clinical effectiveness was evaluated in 235 patients. The FEV_1_ increase from baseline was maintained over the whole study period (+300 mL at 4 months and 1 year, +200 mL at 2 and 3 years, all p < 0.0001). The frequency of exacerbations decreased, and the Asthma Control Test score increased from baseline clinically significantly at all timepoints (+4.3 at 4 months, +4.9 points at 1 year, +5.3 points at 2 years, and +4.6 points at 3 years, all p < 0.0001). Dupilumab was well tolerated; < 10% of patients reported adverse events or discontinued dupilumab treatment. The only adverse events recorded in more than 1 patient were conjunctivitis (4 patients), joint pain (3 patients), and flu-like symptoms (2 patients). Conclusion: In this large real-life study of dupilumab in patients with severe asthma dupilumab showed long-term clinical effectiveness for up to 3 years, with results consistent with prior randomized controlled trials.

## Introduction 

Biologics, monoclonal antibodies directed at key mediators of the inflammatory cascade underlying the asthma pathogenesis, are a well-established maintenance therapy in patients with severe asthma uncontrolled on optimal inhaled treatment. Six monoclonal antibodies have shown clinical efficacy in phase 3 randomized controlled trials (RCTs) [1, 2, 3, 4, 5, 6]. Omalizumab, an anti-immunoglobulin E monoclonal antibody and the first biologic in severe asthma, has been used to treat patients with severe allergic asthma for more than 20 years [1]. The next target for biologic therapy in the inflammatory cascade was interleukin (IL)-5. Mepolizumab [2, 7, 8, 9] and reslizumab [3, 10] are monoclonal antibodies against IL-5, and benralizumab [4, 11, 12, 13] targets the IL-5 receptor. Tezepelumab is a monoclonal antibody against thymic stromal lymphopoietin, a cytokine more upstream in the inflammatory cascade [5, 14, 15]. Dupilumab, an anti-IL-4 receptor α monoclonal antibody, inhibits signaling of IL-4 and IL-13 [6, 16]. 

In phase 3 RCTs, patients receiving dupilumab had a lower rate of severe asthma exacerbations than those receiving placebo, together with clinically relevant improvements in asthma control and lung function [6]. In addition, in RCTs, dupilumab treatment enabled a reduction in oral corticosteroid (OCS) use in patients with severe asthma who required OCS maintenance therapy [16]. However, RCTs recruit carefully selected, relatively narrow patient populations, which typically receive intensive follow-up and care, such that the results (although important) do not necessarily reflect the real-world effectiveness of the treatment tested when used under standard-of-care conditions. Physicians and payers are therefore becoming increasingly interested in data collected under “real-world” conditions, such as those obtained from registries. Real-life studies in patients with severe asthma have confirmed that the effectiveness of dupilumab is similar to the efficacy seen in RCTs [17, 18, 19, 20, 21, 22, 23]. However, although 1 real-life study in patients with severe chronic rhinosinusitis with nasal polyposis (CRSwNP) had a relatively large sample size (n = 648) [24], most real-life studies examining the effectiveness of dupilumab in patients with severe asthma only had a small sample size, and few followed-up patients for more than 1 year. Renner et al. [17] presented retrospective single-center Austrian real-life data from 13 patients with severe asthma receiving dupilumab. Portacci et al. [18] showed prospective single-center Italian real-life data from 18 patients with a follow-up of 1 year. Dupin et al. [19] published the first multi-center real-life data of dupilumab treatment in severe asthma from French asthma centers. In this study, 64 patients were analyzed retrospectively [19]. Castilla-Martínez et al. [21] prospectively observed a multi-center Spanish real-life cohort of 25 patients with severe asthma for 12 weeks. Three studies showed real-life data from cohorts of between 100 and 200 patients with severe asthma [20, 22, 23]. A Dutch center analyzed 148 patients retrospectively [20]. Two Italian multi-center real-life studies evaluated dupilumab treatment in patients with severe asthma [22, 23]. Pelaia et al. [22] analyzed 127 patients retrospectively. Caminati et al. [23] prospectively observed 195 patients for up to 1 year. Among the studies mentioned above, this is the only one reporting prospective real-life data from more than 100 patients, and with a maximum follow-up of 1 year [23]. We therefore aimed to prospectively evaluate the real-life efficacy of dupilumab in a large population of patients with severe asthma from the German Asthma Net (GAN) severe asthma registry over a longer period. 

## Materials and methods 

The severe asthma registry of the GAN is an international registry with centers in Germany and Austria. Patients with asthma from these centers who meet the criteria for severe asthma set by the European Respiratory Society/American Thoracic Society (ERS/ATS) [25], and who provide written informed consent are included in the registry. All data collected until January 1, 2024, were considered in this analysis. 

This real-life, multi-center, non-interventional study aimed to investigate the real-life effectiveness of dupilumab in patients with severe asthma. For this purpose, different patient groups were defined as follows. Baseline characteristics were analyzed in all patients who received at least 1 dose of dupilumab after inclusion into the GAN registry (dupilumab baseline group), using data collected at the last visit prior to dupilumab initiation. Patients who had received dupilumab prior to inclusion in the registry were excluded from this analysis. Patients who had received a biologic other than dupilumab prior to dupilumab initiation were included in the main analyses (dupilumab baseline group and dupilumab follow-up group). Additionally, a separate subgroup analysis of baseline characteristics was performed in those patients (see below). Real-life effectiveness was then assessed in patients of the dupilumab baseline group for whom at least two follow-up visits (4 months and 1 year) were available after initiation of dupilumab treatment (dupilumab follow-up group), so our results are based on an at least 1-year follow-up period. 

Real-life effectiveness was assessed based on asthma control test score (ACT), exacerbations (as defined by the treating physician), lung function (forced expiratory volume in 1 second (FEV_1_)), OCS dose, inhaled corticosteroid (ICS) dose, and fraction of exhaled nitric oxide (FeNO). High-dose ICS was defined according to Global Initiative for Asthma (GINA) [26]. In addition, the peripheral blood eosinophil count (BEC) was assessed longitudinally. Effectiveness was evaluated at 4 months, and 1, 2 and 3 years after initiation of dupilumab. We also assessed how many patients had improvements fulfilling the following definition of clinical remission: no OCS use, no exacerbations, ACT ≥ 20 points, and stable lung function defined as either FEV_1_ ≥ 80% or no decline > 5% compared to baseline. 

Separate analyses of baseline characteristics were conducted to compare patients who switched from other biologics to dupilumab and patients who switched to other biologics after long-term (i.e., at least 2 years) dupilumab treatment. Adverse events and possible treatment discontinuation due to adverse events were documented and are listed descriptively. 

The study was conducted in accordance with the Declaration of Helsinki and approved by independent ethics committees of the German and Austrian centers. 

The data are reported as mean ± standard deviation (SD), or median and interquartile ranges (IQR). Categorical data are presented as percentages and/or numbers. For normally distributed data, Student’s t-test was used to compare baseline and follow-up visits. For non-normally distributed data, Wilcoxon signed-rank test was used. For intergroup comparisons of categorical data, χ^2^-test was used, whereas for quantitative data Mann–Whitney U test was used to compare two groups, and Kruskal–Wallis test was used to compare more than two groups. 

## Results 

On entry into the GAN registry, of 2,919 patients with severe asthma 1,139 (39.0%) had uncontrolled asthma based on GINA control status [26], and measurable evidence of type 2 inflammation defined as BEC ≥ 150 cells/µL and/or FeNO ≥ 25 ppb, and were therefore potentially eligible for dupilumab treatment (Supplemental Table S1). 

A total of 481 patients were included in the dupilumab baseline group, most of whom were adult (n = 457, 95%), with a mean age at initiation of dupilumab treatment of 51.9 years (Table 1a). More patients were female (n = 265, 55%). The median BEC was 174 cells/µL and the median FeNO 47 ppb. Only 56 adult patients (12.3%) had comorbid atopic dermatitis, compared to 8 pediatric patients (34.8%), whereas almost half of the adult patients had CRSwNP (n = 204, 44.6%) compared to 1 pediatric patient. Most of the recruited patients were non-smokers; 184 patients (38.3%) were former smokers, with a median smoking history of 9.6 pack-years. Half of the adult patients had received previous biologic treatments compared to 6 pediatric patients (26.1%). The most common prior biologics were benralizumab (n = 97, 22.1%), mepolizumab (n = 84, 19.1%), and omalizumab (n = 70, 15.9%). 

A total of 235 of these patients had at least 2 available follow-up visits and were therefore included in the dupilumab follow-up cohort. In this group, mean (SD) FEV_1_ improvements vs. baseline were both statistically and clinically significant at 4 months (+300 mL), 1 year (+ 300 mL), 2 years (+ 200 mL), and 3 years (+ 200 mL; all p < 0.0001) (Figure 1). The frequency of exacerbations decreased from baseline in 71.3% of patients at 4 months, 70.0% at 1 year, 60.8% at 2 years, and 43.8% at 3 years (Table 2). Asthma control, as measured by the ACT, increased (i.e., improved) statistically and clinically significantly [27] from baseline by a mean of 4.3 points at 4 months, 4.9 points at 1 year, 5.3 points at 2 years, and 4.6 points at 3 years (all p < 0.0001) (Figure 2). 

The proportion of patients using high-dose ICS decreased significantly after the initiation of dupilumab treatment at all visits, as did the proportion of patients requiring maintenance OCS (p < 0.05 for all) (Table 3). In terms of the biomarkers of type 2 inflammation, FeNO significantly decreased from baseline at all follow-up visits (Table 4a); BEC increased after initiation of dupilumab treatment, with the changes statistically significant at 4 months and 1 year, and decreased thereafter towards the baseline values (Table 4b). 

At year 1, when excluding patients with missing data, 39.2% of patients met all criteria of remission (Supplemental Table S2). When including cases with missing data as “no remission” or “criterion not met”, the percentage was 32.2% (Supplemental Table S3). 

Overall, dupilumab was well tolerated. In the dupilumab follow-up group, few patients reported adverse events (4.3% at 4 months, 2.6% at 1 year, 2.8% at 2 years, 1.4% at 3 years). Similarly, few patients discontinued dupilumab treatment (0.4% at 4 months, 3.5% at 1 year, 4.2% at 2 years, 4.1% at 3 years). Two patients discontinued dupilumab treatment due to asymptomatic but persistently high BEC (> 3,000 cells/µL) (Table 5) (Supplemental Table S4). 

Baseline characteristics before dupilumab treatment initiation did not differ significantly between patients started on dupilumab after having first received another biologic (n = 96), patients who received dupilumab as the initial biologic (n = 285), and patients who received any biologic other than dupilumab as a control group (n = 1,376). Only baseline asthma control measured by ACT was statistically significantly higher in patients started on dupilumab after having first received another biologic compared to patients who received any biologic other than dupilumab, but this difference was not clinically meaningful (Table 1b) [27]. 

## Discussion 

Dupilumab treatment was initiated in 481 of the 2,919 patients with severe asthma included in the GAN registry. These patients constituted 42.3% of the 1,137 patients in the GAN registry with uncontrolled asthma and measurable type 2 inflammation, who were therefore eligible for dupilumab according to GINA recommendations and licensed indication [28]. 

Our data show that dupilumab treatment resulted in clinically significant improvements in asthma control, lung function, exacerbation frequency, and OCS use in these patients. Evidence of the real-life effectiveness is important to complement efficacy data from RCTs, since patients with severe asthma recruited into RCTs do not necessarily adequately reflect “real-life” patients, especially regarding smoking history and comorbidities. To our knowledge, this is to date the largest real-life study to evaluate the effectiveness of dupilumab in patients with severe asthma. 

Comparing the patients in our cohort to the trial population of the dupilumab phase III LIBERTY ASTHMA QUEST study [6], our patients were more likely to be former smokers (38.3. vs. 19.3%) with a more intensive smoking history (median 9.6 pack-years vs. mean 4.02 pack-years). Furthermore, median baseline BEC was higher in the phase III study population (255 cells/µL) than in our cohort (174 cells/µL). These differences highlight the importance of our study, as they confirm a highly selected phase III trial population with a strong type 2 signal. Phase III results also indicate that the efficacy of dupilumab increases with increasing BEC. 

Nonetheless, clinical outcomes were comparable in our real-life and the LIBERTY ASTHMA QUEST cohort. Mean increases in FEV_1_ at 4 months and 1 year (both +300 mL) were similar to the increases in the 300-mg group of the phase III study, the now approved dose. A direct comparison of the improvements of asthma control was not possible, as our study used the ACT and the LIBERTY ASTHMA QUEST study the 5-item asthma control questionnaire (ACQ-5). The rationale for using the ACT in the GAN registry was the longer recall time of 4 weeks compared to 1 week in the ACQ-5, as well as the ACT being the more commonly used questionnaire in Austria and Germany. Further, since in our registry documentations of biologic treatment are annual (with the exception of the 4-month visit after initiation), the ACT should provide a more robust estimation of long-term asthma control. At the 1-year visit, mean ACT improved by 4.9 points (1.63 times the MCID of 3 points [27]) in our study, compared to a mean ACQ-5 improvement of 1.52 points (3.04 times the MCID of 0.5 points [29]) at 52 weeks in the 300-mg dupilumab group of the LIBERTY ASTHMA QUEST study. 

Long-term outcomes are particularly important in severe asthma [30, 31]. The TRAVERSE open-label extension study demonstrated the long-term efficacy of dupilumab in a highly selected population for up to 148 weeks [32]. In our real-life study the clinically improvements mentioned above were observed at all timepoints (4 months and 1, 2, and 3 years), making this the first study to show real-life effectiveness similar to what was seen in RCTs well beyond 1 year. Mean increase in FEV_1_ at 3 years (+200 mL) is clinically significant but slightly lower than in the TRAVERSE study for patients who received dupilumab for the full 148 weeks recruited from the LIBERTY ASTHMA QUEST study (+330 mL) and for 110 weeks recruited from the phase 2b study (+220 mL) [6, 33]. 

In a recent meta-analysis [34] 30 – 38% of the patients with severe asthma attained remission under biologic treatment. The assessment of remission was performed at 12 months in a majority of the studies included in that meta-analysis. The remission data from our analysis are in line with the results of the meta-analysis, and confirm both the principal and long-term impact of dupilumab on relevant outcomes reflecting clinical efficacy in a large real-world patient population. 

It is important to note that approximately 50% of patients in our study were biologic pre-treated, representing a more difficult-to-treat cohort. The results of the study demonstrated that dupilumab treatment was not only effective but also well tolerated. Out of 235 patients with at least two follow-up visits, only 21 adverse events were reported. Of these, only 4 led to treatment discontinuations, with a total of only 18 treatment discontinuations reported. Equally important, as expected due to prevention of blood eosinophil trafficking into tissue, transient increases in blood eosinophil counts with dupilumab were observed, but resulted in dupilumab discontinuation in only 2 out of 481 patients. Our analyses also strongly suggest that eosinophil numbers tend to return to baseline values over time. 

The key strengths of this study are its position as the largest real-life dupilumab study in patients with severe asthma to date and the use of long-term data (available for up to 3 years). The study is limited by being based on registry data. Also, at 2 and 3 years there is a drop in patient numbers. This is not due to treatment discontinuation but rather due to continuous enrolment in the registry. 

## Conclusion 

Dupilumab demonstrated effectiveness in real-life patients with severe asthma, similar to the results in selected populations in RCTs. This effectiveness was sustained over many years. Furthermore, dupilumab was well tolerated. 

## Authors’ contributions 

Andreas Renner: conception, data collection, data analysis, writing (original draft); Slagjana Stoshikj: data collection, writing (revising); Eckard Hamelmann: data collection, writing (revising); Dirk Skowasch: data collection, writing (revising); Christian Schulz: data collection, writing (revising); Margret Jandl: data collection, writing (revising); Olaf Schmidt: data collection, writing (revising); Katrin Milger: data collection, writing (revising); Rainer Ehmann: data collection, writing (revising); Christian Taube: data collection, writing (revising); Stephanie Korn: data collection, writing (revising); Roland Buhl: conception, data collection, writing (revising); Marco Idzko: conception, data collection, writing (revising). 

## Funding 

This study was funded by Sanofi-Aventis. Statistical support was provided by Annette Holtdirk (RQM+ (Germany), Hamm, Germany, and was funded by Sanofi-Aventis Deutschland GmbH, Germany. Medical writing support was provided by David Young (Young Medical Communications and Consulting Ltd), Horsham, United Kingdom. 

## Conflict of interest 

AR has no conflicts of interest to report. 

SS reports speaker fees from AstraZeneca, Sanofi, all outside the submitted work. 

EH is funded by the German Ministry of Education and Research (BMBF) as consortional partner in CHAMP [01GL1742D] for research in severe asthma in children. 

DS received fees for lectures or consultations from AstraZeneca, Bayer, Boehringer Ingelheim, Chiesi, GSK, Janssen, Novartis, Pfizer. 

CS reports speaker fees from AstraZeneca and Novartis all outside the submitted work. 

MJ has nothing to disclose. 

OS reports speaker fees from AstraZeneca, GSK, Sanofi, and Boehringer all outside the submitted work. 

KM reports speaker fees from AstraZeneca, GSK, Novartis, Sanofi, all outside the submitted work. 

RE has nothing to disclose. 

CT has nothing to disclose. 

SK reports speaker fees from AstraZeneca, GSK, Novartis, Sanofi, all outside the submitted work. 

RB reports grants to Mainz University and personal fees from Boehringer Ingelheim, GSK, Novartis, and Roche, as well as personal fees from AstraZeneca, Celltrion, Chiesi, Cipla, Sanofi, and Teva, all outside the submitted work. 

MI reports lecture fees from AstraZeneca, Bayer, Berlin-Chemie, Boehringer Ingelheim, Chiesi, CSL-Behring, GSK, Menarini, MSD, Novartis, Roche, Sanofi, and Thermofischer; and advisory board fees from Alk-Pharma, AstraZeneca, Berlin-Chemie, Boehringer Ingelheim, Chiesi, CSL-Behring, GSK, Novartis, and Sanofi, all outside the submitted work. 

**Table 1a.** Baseline characteristics of dupilumab patients.

		**Age (years)**		**Total (n = 481)**
**Item**		**< 18 (n = 24)**	**≥ 18 (n = 457)**	
Age (years)	Mean (SD)	12.5 (3.2)	54.0 (12.3)	51.9 (15.0)
Age at asthma diagnosis (years)	Mean (SD)	4.8 (4.6)	34.2 (18.7)	33.2 (19.1)
Age at asthma diagnosis	Before 12 years	22 (91.7%)	100 (21.9%)	122 (25.4%)
	After 12 years	2 (8.3%)	357 (78.1%)	359 (74.6%)
BMI (kg/m^2^)	Mean (SD)	21.2 (5.1)	28.3 (6.2)	27.9 (6.4)
Smoking habits	Non-smoker	24 (100%)	262 (57.3%)	286 (59.5%)
	Smoker	–	11 (2.4%)	11 (2.3%)
	Former smoker	–	184 (40.3%)	184 (38.3%)
If smoker, pack-years	N		11	11
	Median (IQR)		4.5 (1.5 – 15.0)	4.5 (1.5 – 15.0)
If former smoker, pack-years	N		184	184
	Median (IQR)		9.6 (4.3 – 23.3)	9.6 (4.3 – 23.3)
Nasal polyps	Yes	1 (4.3%)	204 (44.6%)	205 (42.7%)
Chronic sinusitis	Yes	–	220 (48.1%)	220 (45.8%)
Any allergic comorbidities	Yes	21 (91.3%)	274 (60.0%)	295 (61.5%)
	Atopic eczema	8 (34.8%)	56 (12.3%)	64 (13.3%)
	Food allergy	4 (17.4%)	85 (18.6%)	89 (18.5%)
PBEC (cells/µL)	Median (IQR)	533.0 (0.7 – 990.0)	171.6 (62.4 – 406.4)	174.0 (61.2 – 436.0)
FeNO at presentation day (ppb)	Median (IQR)	23.0 16.0 – 40.0)	49.0 (25.0 – 85.5)	47.0 (25.0 – 83.0)
ACT (points)	Mean (SD)	17.0 (5.6)	13.5 (5.1)	13.6 (5.2)
FEV_1_ (L)	Mean (SD)	2.4 (0.8)	2.1 (0.8)	2.1 (0.8)
FEV_1_ (% predicted)	Mean (SD)	86.2 (12.7)	65.5 (21.8)	66.4 (21.9)
Exacerbations	None	3 (12.5%)	36 (7.9%)	39 (8.1%)
	Unknown	3 (12.5%)	54 (11.8%)	57 (11.9%)
	1×/year	4 (16.7%)	65 (14.2%)	69 (14.3%)
	> 1×/year, but < 1×/month	13 (54.2%)	236 (51.6%)	249 (51.8%)
	≥ 1×/month	1 (4.2%)	66 (14.4%)	67 (13.9%)
GINA control status	Controlled	5 (20.8%)	36 (7.9%)	41 (8.5%)
	Partly controlled	7 (29.2%)	58 (12.7%)	65 (13.5%)
	Uncontrolled	12 (50.0%)	363 (79.4%)	375 (78.0%)
Other biologics in the past	Yes	6 (26.1%)	208 (50.0%)	214 (48.7%)
Previous biologics (multiple entries)	Benralizumab	–	97 (23.3%)	97 (22.1%)
	Mepolizumab	4 (17.4%)	80 (19.2%)	84 (19.1%)
	Omalizumab	4 (17.4%)	66 (15.9%)	70 (15.9%)
	Reslizumab	–	7 (1.7%)	7 (1.6%)
	Tezepelumab	–	5 (6.3%)	5 (5.7%)

ACT = asthma control test; PBEC = peripheral blood eosinophil count; FeNO = fraction of exhaled nitric oxide; SD = standard deviation; IQR = interquartile range.

**Table 1b.** Comparison of baseline characteristics of patients started on dupilumab after having first received another biologic, patients who received dupilumab as the initial biologic, and patients who received any biologic other than dupilumab.

		**Groups**			**Comparison**		
**Item**		**Group 1 Dupilumab after other biologic**	**Group 2 Only dupilumab**	**Group 3 Any other biologic**	**Group 1 vs. 2**	**Group 1 vs. 3**	**All groups**
Systemic steroids	N	96	285	1378			
	No	67 (69.8%)	221 (77.5%)	976 (70.8%)	0.1262^1^	0.8292^1^	0.0645^1^
	Yes	29 (30.2%)	64 (22.5%)	402 (29.2%)			
Prednisolone equivalent dose (mg)	N	96	285	1368			
	Mean (SD)	2.3 (5.4)	3.1 (10.5)	3.6 (9.4)	0.2063^2^	0.9174^2^	0.0932^3^
GINA control status	N	96	285	1376			
	Controlled	24 (25.0%)	80 (28.1%)	314 (22.8%)	0.1999^1^	0.1389^1^	0.1146^1^
	Partly controlled	37 (38.5%)	82 (28.8%)	422 (30.7%)			
	Uncontrolled	35 (36.5%)	123 (43.2%)	640 (46.5%)			
ACT (points)	N	92	264	1,248			
	Mean (SD)	18.0 (5.3)	17.3 (5.6)	16.5 (5.7)	0.3249^2^	0.0215^2^	0.0172^3^
FEV1 [L]	N	86	273	1301			
	Mean (SD)	2.27 (0.92)	2.27 (0.83)	2.14 (0.86)	0.8255^2^	0.2207^2^	0.0182^3^
FEV1 [%]	N	85	273	1,289			
	Mean (SD)	70.8 (22.7)	74.4 (22.4)	71.4 (22.2)	0.2001^2^	0.8310^2^	0.0946^3^
Symptoms during the day	N	96	284	1376			
	Never	9 (9.4%)	27 (9.5%)	129 (9.4%)	0.7257^1^	0.2248^1^	0.2526^1^
	≤ 2×/week	32 (33.3%)	74 (26.1%)	339 (24.6%)			
	> 2×/week, but < 1×/day	25 (26.0%)	80 (28.2%)	324 (23.5%)			
	≥ 1×/day	26 (27.1%)	91 (32.0%)	496 (36.0%)			
	unknown	4 (4.2%)	12 (4.2%)	88 (6.4%)			
Symptoms during the night	N	96	285	1,376			
	Never	42 (43.8%)	92 (32.3%)	443 (32.2%)	0.1823^1^	0.0625^1^	0.1178^1^
	≤ 2×/week	10 (10.4%)	54 (18.9%)	214 (15.6%)			
	> 2×/week, but < 1×/day	10 (10.4%)	32 (11.2%)	166 (12.1%)			
	≥ 1×/day	18 (18.8%)	47 (16.5%)	194 (14.1%)			
	Common nocturnal symptoms	11 (11.5%)	48 (16.8%)	283 (20.6%)			
	Unknown	5 (5.2%)	12 (4.2%)	76 (5.5%)			

^1^χ^2^-test; ^2^Mann-Whitney U-test; ^3^Kruskal-Wallis test. ACT = asthma control test; PBEC = peripheral blood eosinophil count; FeNO = fraction of exhaled nitric oxide; SD = standard deviation.

**Figure 1:**
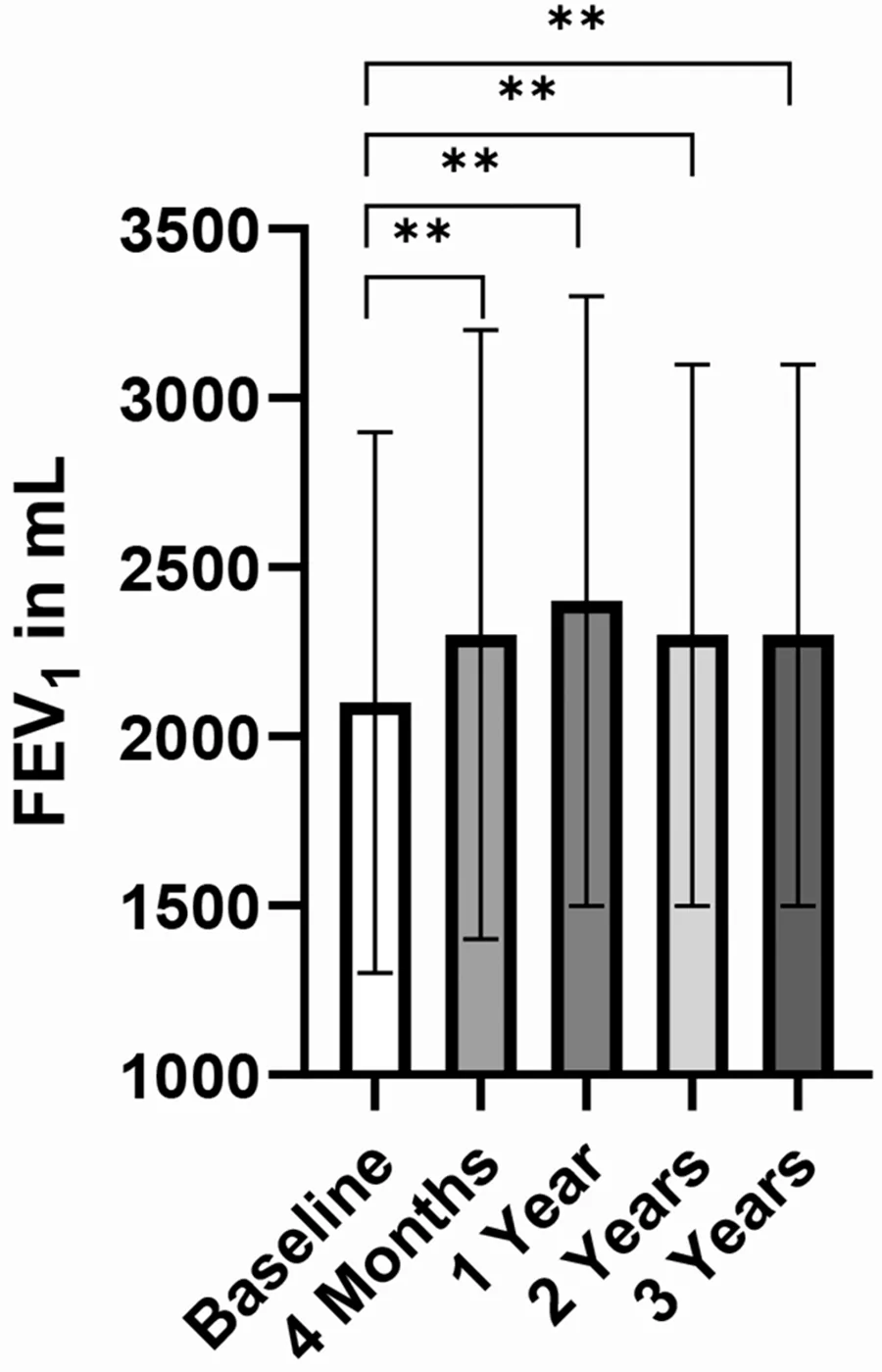
Forced expiratory volume in 1 second (FEV_1_) in mL at baseline (2,100 ± 800) and follow-up visits (month 4 2,300 ± 900, year 1 2,400 ± 900, year 2 2,300 ± 800, year 3 2,300 ± 800). Bars indicate mean, whiskers standard deviation. Statistical significances of differences between means were calculated using Student’s t-test. **p < 0.0001.

**Figure 2:**
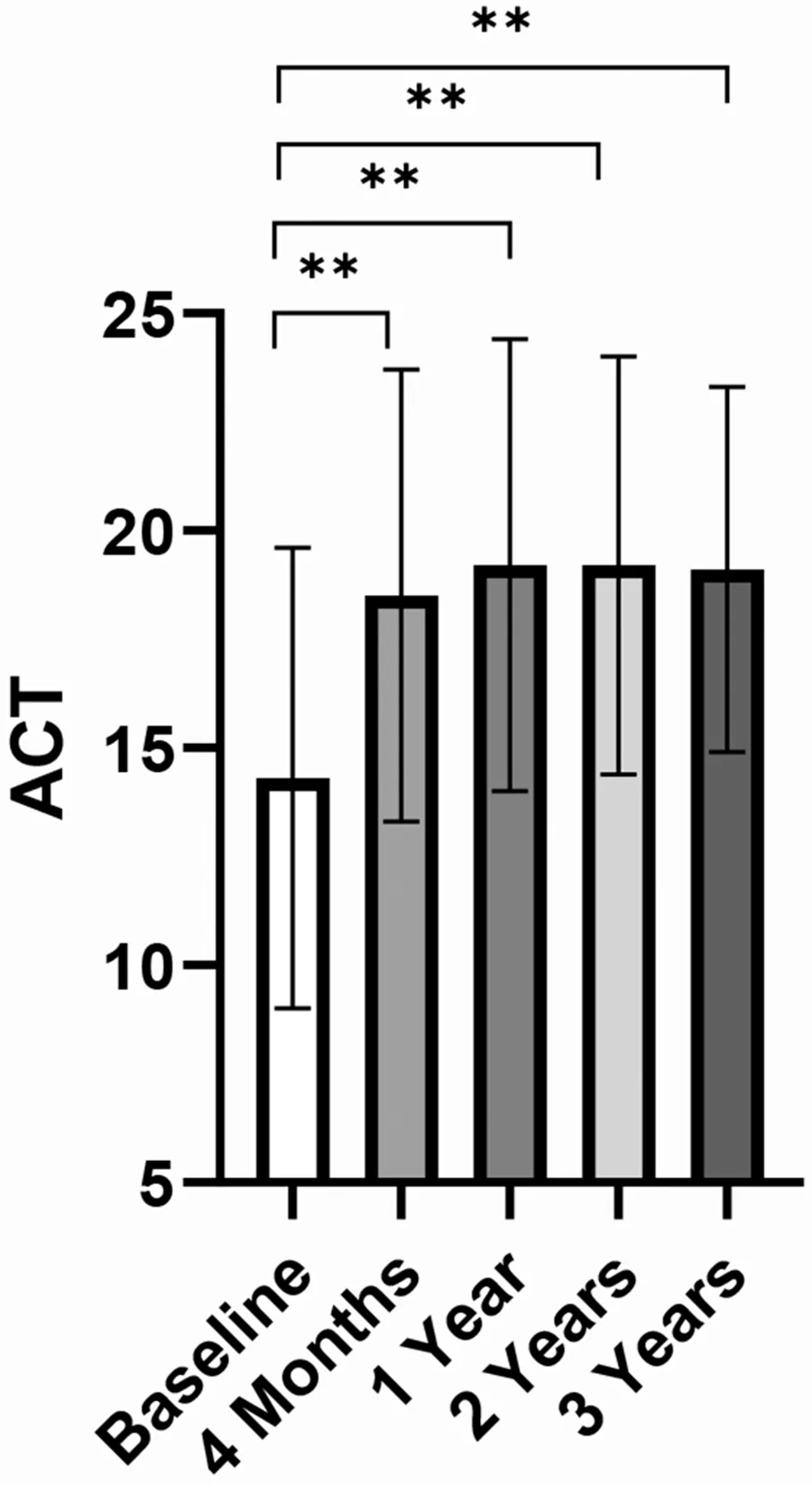
Asthma control test (ACT) at baseline (14.3 ± 5.3) and follow-up visits (month 4 18.5 ± 5.2, year 1 19.2 ± 5.2, year 2 19.2 ± 4.8, year 3 19.1 ± 4.2). Bars indicate mean, whiskers standard deviation. Statistical significances of differences between means were calculated using Student’s t-test. **p < 0.0001.

**Table 3.** Real-life effectiveness of dupilumab on inhaled and systemic steroid use.

		**Visit**				
**Item**		**Baseline (n = 235)**	**Month 4 (n = 230)**	**Year 1 (n = 230)**	**Year 2 (n = 143)**	**Year 3 (74)**
High-dose ICS+LABA (+LAMA if necessary)	Yes	213 (90.6%)	192 (83.5%)	180 (78.3%)	115 (80.4%)	60 (82.2%)
High-dose ICS+LABA (+LAMA if necessary) - Changes from baseline	Discontinued		19 (8.3%)	32 (13.9%)	19 (13.3%)	10 (13.7%)
	Unchanged		19 (8.3%)	18 (7.8%)	9 (6.3%)	3 (4.1%)
	Changed		189 (82.2%)	176 (76.5%)	111 (77.6%)	58 (79.5%)
	Newly added		3 (1.3%)	4 (1.7%)	4 (2.8%)	2 (2.7%)
	Sign test (p-value)		0.0009	< 0.0001	0.0026	0.0386
Systemic steroids	Yes	90 (38.3%)	63 (27.4%)	46 (20.0%)	18 (12.6%)	7 (9.6%)
Systemic steroids – Changes from baseline	Discontinued		30 (13.0%)	51 (22.2%)	36 (25.2%)	18 (24.7%)
	Unchanged		137 (59.6%)	133 (57.8%)	89 (62.2%)	48 (65.8%)
	Changed		57 (24.8%)	37 (16.1%)	18 (12.6%)	6 (8.2%)
	Newly added		6 (2.6%)	9 (3.9%)	–	1 (1.4%)
	Sign test (p-value)		< 0.0001	< 0.0001	< 0.0001	< 0.0001
Prednisolone equivalent dose (mg)	Median (IQR)	0.0 (0.0 – 5.0)	0.0 (0.0 – 2.5)	0.0 (0.0 – 0.0)	0.0 (0.0 – 0.0)	0.0 (0.0 – 0.0)
Prednisolone equivalent dose (mg) – Changes from baseline	Median (IQR)		0.0 (–2.5 – 0.0)	0.0 (–5.0 – 0.0)	0.0 (–5.0 – 0.0)	0.0 (–5.0 – 0.0)
	Signed Rank test (p-value)		< 0.0001	< 0.0001	< 0.0001	< 0.0001
Prednisolone equivalent dose (mg) – Changes from baseline	Discontinued		30 (13.1%)	51 (22.2%)	36 (25.2%)	18 (24.7%)
	Dose ≤ 50% of baseline		23 (10.0%)	14 (6.1%)	8 (5.6%)	3 (4.1%)
	Dose > 50% of baseline		34 (14.8%)	23 (10.0%)	10 (7.0%)	3 (4.1%)
	Unchanged		137 (59.8%)	133 (57.8%)	89 (62.2%)	48 (65.8%)
	Newly added		5 (2.2%)	9 (3.9%)	–	1 (1.4%)

IQR = interquartile range; ICS = inhaled corticosteroid; LABA = long-acting β2-agonist; LAMA = long-acting muscarinic antagonist.

**Table 2.** Real-life effectiveness of dupilumab on exacerbation frequency.

		**Visit**				
**Item**		**Baseline (n = 235)**	**Month 4 (n = 230)**	**Year 1 (n = 230)**	**Year 2 (n = 143)**	**Year 3 (74)**
Exacerbations	None	21 (8.9%)	–	–	–	–
	Unknown	17 (7.2%)	–	–	–	–
	1×/year	35 (14.9%)	–	–	–	–
	> 1×/year, but < 1×/month	127 (54.0%)	–	–	–	–
	≥ 1×/month	35 (14.9%)	–	–	–	–
Change in exacerbation frequency from baseline	Decreased		164 (71.3%)	161 (70.0%)	87 (60.8%)	32 (43.8%)
	Unchanged		35 (15.2%)	59 (25.7%)	50 (35.0%)	36 (49.3%)
	Increased		4 (1.7%)	10 (4.3%)	6 (4.2%)	5 (6.8%)
	Not yet assessable		27 (11.7%)	–	–	–

**Table 4a.** Changes in fraction of exhaled nitric oxide after initiation of dupilumab treatment.

		**Visit**				
**Item**		**Baseline (n = 235)**	**Month 4 (n = 230)**	**Year 1 (n = 230)**	**Year 2 (n = 143)**	**Year 3 (74)**
FeNO (ppb)	N	167	153	131	66	27
	Median (IQR)	45.0 (24.0 – 79.0)	20.0 (13.0 – 32.0)	21.0 (15.0 – 29.0)	21.5 (14.0 – 29.0)	19.0 (11.0 – 30.0)
FeNO (ppb) – Changes from baseline	N		133	111	57	20
	Median (IQR)		–19.0 (–53.0 – –3.0)	–24.0 (–55.0 – –3.0)	–27.0 (-52.0 – –4.0)	–22.5 (–45.0 – –7.5)
	Signed rank test (p-value)		< 0.0001	< 0.0001	< 0.0001	0.0001

FeNO = fraction of exhaled nitric oxide; IQR = interquartile range.

**Table 4b.** Changes in peripheral blood eosinophil count after initiation of dupilumab treatment.

		**Visit**				
**Item**		**Baseline (n = 235)**	**Month 4 (n = 230)**	**Year 1 (n = 230)**	**Year 2 (n = 143)**	**Year 3 (74)**
PBEC (cells/µL)	N	146	122	111	67	34
	Median	172.8	306.8	290.0	240.6	196.0
	IQR	61.0 – 386.4	140.0 – 590.0	129.2 – 540.0	120.0 – 360.0	104.0 – 270.0
Changes in PBEC (cells/µL) from baseline	N		111	91	57	31
	Median		47.1	48.9	2.0	30.7
	IQR		–80.7 – 391.4	–94.0 – 317.0	–169.1 – 231.4	–227.4 – 180.0
	Signed rank test (p-value)		0.0003	0.0031	0.4529	0.9238

PBEC = peripheral blood eosinophil count; IQR = interquartile range.

**Table 5.** Listing of discontinuations.

**Pat. ID**	**Visit**	**Therapy is continued**	**Reason for discontinuation**
2042	Year 1	No	Patient wish, subjective worsening of food intolerances
2178	Year 3	No	Inadequate clinical response
2197	Year 2	No	Inadequate clinical response
2723	Year 1	No	No clinical response, worsening of atopic dermatitis
2802	Year 2	No	Joint pain
2885	Year 2	No	No clinical response
3017	Year 3	No	ICU stay with ECMO due to COVID-ARDS
3097	Year 1	No	No clinical response
3122	Year 1	No	Flu- like symptoms and palpitations
3168	Year 1	No	Treatment change to tezepelumab due to lack of clinical response
3193	Year 1	No	No clinical response
3289	Year 2	No	Flu-like symptoms for 6 months
3479	Year 3	No	Patient wish
3714	Month 4	No	Psoriasis
4183	Year 1	No	Treatment change to omalizumab by an ENT specialist
4280	Year 1	No	Permanently elevated peripheral blood eosinophil count
4330	Year 2	No	No clinical response
4498	Year 2	No	Permanently elevated peripheral blood eosinophil count

## Supplemental material

Supplemental material Dupilumab improves asthma control in German severe asthma patients
