# Supplementary material for: Dupilumab improves long-term asthma control in German patients with severe asthma: Real-world data
Source: Allergol Select. 2026 Sep 25;10:268–79. doi: 10.5414/ALX02646E (PMC13647249; doi:10.5414/ALX02646E)
Supplement: Supplementary file 1 [file allergologieselect-10-268-S01.pdf]

Dupilumab improves asthma control in German severe asthma patients

Supplementary material

Table S1: Selected baseline characteristics of all patients included in the GAN-registry

		Age [years]		
**Item**		**<18 (n=245)**	**≥ 18 (n=2674)**	**Total (n=2919)**
Number of patients with severe asthma	N	245	2674	2919
GINA control status	N	245	2674	2919
	controlled	68 (27.8%)	330 (12.3%)	398 (13.6%)
	partly controlled	104 (42.4%)	611 (22.8%)	715 (24.5%)
	uncontrolled	59 (24.1%)	1725 (64.5%)	1784 (61.1%)
Uncontrolled and Type-2-inflammation*	N	245	2674	2919
	no/unknown	221 (90.2%)	1559 (58.3%)	1780 (61.0%)
	uncontrolled, Type 2	24 (9.8%)	1115 (41.7%)	1139 (39.0%)
PBEC [cells/µL]	N	74	1688	1762
	Median	278.22	250.00	250.00
	IQR	60.00-580.00	100.58-501.54	100.00-505.80
PBEC ≥ 150/µL	N	46 (62.2%)	1090 (64.6%)	1136 (64.5%)
FeNO [ppb]	N	116	1442	1558
	Mean (SD)	28.13 (27.01)	50.04 (49.05)	48.41 (48.10)
FeNO ≥ 25 ppb	N	39 (33.6%)	917 (63.6%)	956 (61.4%)

*measurable evidence of type 2 inflammation defined as PBEC ≥ 150 cells/µL and/or FeNO ≥ 25 ppb. PBEC, peripheral blood eosinophil count; FeNO, fraction of exhaled nitric oxide; SD, standard deviation; IQR, interquartile range.

Table S2: Remission excluding patients with missing data					
**Time**	**Remission criterion**	**N total**	**N**	**[%]**	**95% Confidence interval (Clopper-Pearson (Exact)**
Month 4	OCS	230	167	72.6	(66.4% - 78.3%)
	EXA	230	168	73.0	(66.8% - 78.7%)
	ACT	198	100	50.5	(43.3% - 57.7%)
	FEV1	214	189	88.3	(83.2% - 92.3%)
	EXA + ACT	198	76	38.4	(31.6% - 45.5%)
	EXA + ACT + FEV1	189	70	37.0	(30.1% - 44.3%)
	**All criteria**	**189**	**59**	**31.2**	**(24.7% - 38.3%)**
Year 1	OCS	230	184	80.0	(74.2% - 85.0%)
	EXA	230	164	71.3	(65.0% - 77.1%)
	ACT	202	117	57.9	(50.8% - 64.8%)
	FEV1	214	190	88.8	(83.8% - 92.7%)
	EXA + ACT	202	95	47.0	(40.0% - 54.2%)
	EXA + ACT + FEV1	189	85	45.0	(37.7% - 52.4%)
	**All criteria**	**189**	**74**	**39.2**	**(32.2% - 46.5%)**
Year 2	OCS	143	125	87.4	(80.8% - 92.4%)
	EXA	143	94	65.7	(57.3% - 73.5%)
	ACT	121	64	52.9	(43.6% - 62.0%)
	FEV1	129	116	89.9	(83.4% - 94.5%)
	EXA + ACT	121	49	40.5	(31.7% - 49.8%)
	EXA + ACT + FEV1	108	39	36.1	(27.1% - 45.9%)
	**All criteria**	**108**	**33**	**30.6**	**(22.1% - 40.2%)**
	OCS	73	66	90.4	(81.2% - 96.1%)

Year 3	EXA	73	38	52.1	(40.0% - 63.9%)
	ACT	72	37	51.4	(39.3% - 63.3%)
	FEV1	67	61	91.0	(81.5% - 96.6%)
	EXA + ACT	72	26	36.1	(25.1% - 48.3%)
	EXA + ACT + FEV1	66	21	31.8	(20.9% - 44.4%)
	**All criteria**	**66**	**21**	**31.8**	**(20.9% - 44.4%)**

EXA: No exacerbations in the previous year; OCS: no need for systemic corticosteroids in the previous year; ACT: ACT >= 20; FEV1: stable lung function defined as either FEV_1 >= 80% or no decline > 5%compared to baseline. If a criterion is not available/missing, the case is not included in the calculation of the percentages.

Table S3: Remission including patients with missing data					
**Time**	**Remission criterion**	**N total**	**N**	**[%]**	**95% Confidence interval (Clopper-Pearson (Exact)**
Month 4	OCS	230	167	72.6	(66.4% - 78.3%)
	EXA	230	168	73.0	(66.8% - 78.7%)
	ACT	230	100	43.5	(37.0% - 50.2%)
	FEV1	230	189	82.2	(76.6% - 86.9%)
	EXA + ACT	230	76	33.0	(27.0% - 39.5%)
	EXA + ACT + FEV1	230	70	30.4	(24.6% - 36.8%)
	**All criteria**	**230**	**59**	**25.7**	**(20.1% - 31.8%)**
Year 1	OCS	230	184	80.0	(74.2% - 85.0%)
	EXA	230	164	71.3	(65.0% - 77.1%)
	ACT	230	117	50.9	(44.2% - 57.5%)
	FEV1	230	190	82.6	(77.1% - 87.3%)
	EXA + ACT	230	95	41.3	(34.9% - 48.0%)
	EXA + ACT + FEV1	230	85	37.0	(30.7% - 43.5%)
	**All criteria**	**230**	**74**	**32.2**	**(26.2% - 38.6%)**
Year 2	OCS	143	125	87.4	(80.8% - 92.4%)
	EXA	143	94	65.7	(57.3% - 73.5%)
	ACT	143	64	44.8	(36.4% - 53.3%)
	FEV1	143	116	81.1	(73.7% - 87.2%)
	EXA + ACT	143	49	34.3	(26.5% - 42.7%)
	EXA + ACT + FEV1	143	39	27.3	(20.2% - 35.3%)
	**All criteria**	**143**	**33**	**23.1**	**(16.4% - 30.9%)**
Year 3	OCS	73	66	90.4	(81.2% - 96.1%)
	EXA	73	38	52.1	(40.0% - 63.9%)
	ACT	73	37	50.7	(38.7% - 62.6%)

	FEV1	73	61	83.6	(73.0% - 91.2%)
	EXA + ACT	73	26	35.6	(24.7% - 47.7%)
	EXA + ACT + FEV1	73	21	28.8	(18.8% - 40.6%)
	**All criteria**	**73**	**21**	**28.8**	**(18.8% - 40.6%)**

EXA: No exacerbations in the previous year; OCS: no need for systemic corticosteroids in the previous year; ACT: ACT >= 20; FEV1: stable lung function defined as either FEV_1 >= 80% or no decline > 5%compared to baseline. If a criterion is not available/missing, the case is nevertheless included in the calculation of the percentages (as no remission/criterion not met)

Table S4: Adverse events and treatment continuation

		Visit			
**Item**		**Month 4**	**Year 1**	**Year 2**	**Year 3**
Adverse events	N	230	230	143	74
	yes	10 (4.3%)	6 (2.6%)	4 (2.8%)	1 (1.4%)
Treatment continuation	N	230	230	143	74
	no	1 (0.4%)	8 (3.5%)	6 (4.2%)	3 (4.1%)

Table S5 Listing of adverse events			
**Pat.ID**	**Visit**	**Description of adverse events**	**Therapy is continued**
1366	Month 4	Oral herpes, conjunctivits	yes
	Year 1	Oral herpes, conjunctivits	yes
1824	Year 1	Atopic dermatitis	yes
2112	Year 1	Conjunctivitis	yes
2171	Month 4	Migraine, resulting in a dose reduction to 200mg	yes
2190	Year 2	Relapse of preexisting goiter	yes
2411	Year 1	Temporary rash	yes
2757	Month 4	Weight gain	yes
	Year 1	Weight gain	yes
	Year 3	Weight gain	yes
2802	Year 2	Joint pain	no
2983	Month 4	Transient vision loss	yes
3289	Year 2	Flu-like symptoms for 6 months	no
3292	Month 4	Conjunctivitis	yes
3293	Month 4	Conjunctivitis	yes
	Year 1	Conjunctivitis	yes

3480	Month 4	Joint pain and itchy skin	yes
3601	Month 4	Flu-like symptoms once after an application	yes
3714	Month 4	Psoriasis	no
4498	Year 2	Permanently elevated peripheral blood eosinophil count	no
4519	Month 4	Mild intermittent joint pain in the evening	yes
